# Feasibility and cost of partnering with social media influencers to promote colorectal cancer screening

**DOI:** 10.1093/her/cyag024

**Published:** 2026-08-21

**Authors:** Heather Orom, Natasha C Allard, Tiffany Nguyen

**Affiliations:** Department of Community Health and Health Behavior, 304 Kimball Tower, 3435 Main St., University at Buffalo, Buffalo, NY 14214, United States; Department of Community Health and Health Behavior, 304 Kimball Tower, 3435 Main St., University at Buffalo, Buffalo, NY 14214, United States; Georgetown University, 3700 O St., N.W., Washington, DC 20057, United States

## Abstract

There is considerable interest in collaborating with social media influencers to promote health messages, but little evidence demonstrating how this might be done in organizations with few resources or experience. We tested the feasibility and cost of partnering with social media nano- and micro-influencers to promote colorectal cancer (CRC) screening messages. We identified 16 US influencers through an online influencer marketing campaign platform. To test if influencers would create theory-consistent content, we provided them with recommended CRC screening information and guidelines for including one of four message strategies (threat, efficacy, threat and efficacy, information only). Influencers created and posted one video promoting CRC screening on their Instagram Reels and Stories, June–July, 2025. Engagement metrics were collected 1 month after posting. The videos were viewed 89 764 times (M = 5610, SD = 5545), and cost $0.13 per view. They did not elicit resistance; comments were 96% positive, 4% neutral, 0% negative, and engagement rates were high (mean = 7.9%). Influencers were largely able to follow written guidance for including theory-based content in their posts. In light of the rise in social media use, including among middle-aged and older adults, partnerships between public health organizations and social media influencers could expand health message reach.

## Introduction

Colorectal cancer (CRC) is the fourth most common cancer and the second deadliest [1]. Yet there are effective screening modalities for early detection that increase the likelihood of survival, and even prevent cancer through removal of pre-cancerous polyps [2]. CRC screening is recommended for those at average risk between the ages of 45 and 75 years [2]; yet has only a 65% screening adherence rate [1]. A 10-percentage-point increase in screening rates could save an estimated 11 000 lives [3] and would mean the United States would exceed the Healthy People 2030 target of 73% adherence. A key task for public health is to reach the millions of people living in the US who could benefit from CRC screening.

A challenge—and opportunity—for public health communication is to adapt to changes in how people consume information. Over the past decade, media consumption patterns have shifted, with a growing preference for online media due to its accessibility and interactivity, accompanied by a decline in traditional media consumption [4]. Social media use is common even among older adults. For example, US adults 50–64 use most major social media sites at high rates (YouTube 86%, Facebook 70%, Instagram 36%, TikTok 26%) [5]. Even people 65 years and older are using these channels (YouTube 65%, Facebook 59%, Instagram 19%, TikTok 10%) [5]. Among individuals aged 45–49, who have the lowest CRC screening rates [6], social media use is particularly high.

There is CRC-related content available on social media [7, 8], with influencer and celebrity posts having become a notable source. For example, Ryan Reynolds and Rob McElhenney posted their colonoscopies on X in 2023. Celebrity colonoscopies, diagnoses, and deaths can elevate the salience of CRC. In the time period following their colonoscopies, CRC news and social media posts increased in general [9]. Interestingly, the quality of CRC information on social media may also have increased after the Reynold/McElhenney posts, at least temporarily eliminating the quality gap between physician and non-physician care provider TikToks [10]. However, social media content can also spread misinformation. For example, a 2023 study demonstrated that health care provider TikToks are higher quality than non-health care providers’ TikToks, with more of the latter containing claims that are inconsistent with scientific evidence [11]. The same dynamic was noted for YouTube videos, along with an inverse relation between accuracy and number of views [12].

Collaboration is a viable path to increasing influencers’ inclusion of evidence-based information. Influencer training can increase their inclusion of evidence-based information [13]. The involvement of public health professionals in collaborating with social media influencers is potentially a double win. Public health organizations might be able to expand their audience reach by collaborating with social media influencers and raising the quality of public health information available on social media. Indeed, there have been several calls for health promotion and disease prevention to invest in influencer marketing [14, 15]. In addition to having wide reach [16, 17], influencers are persuasive because their followers perceive them as authentic [16, 18, 19], relatable [16], and credible [16, 20], and because they often communicate through personal stories [16] and social norm modeling [16]. These characteristics foster audience processes that increase the likelihood of persuasion, including developing parasocial relationships [21–23], aspirational identification with the influencer [24], trusting the influencer [16, 17], and emotional engagement with the message [16, 23]. These qualities may be important in a communication ecosphere in which people may feel overwhelmed, skeptical of sources, or have non-informational goals for interacting with media such as social connectedness or entertainment [25, 26]. The for-profit sector is spending an increasing proportion of its marketing budgets on influencer campaigns. Half of brands allocate greater than 20% of their marketing budget to influencer marketing [27].

A number of peer-reviewed influencer health campaigns indicate potential for substantial reach, including millions of impressions, number of times the content is displayed on screens, and tens of thousands of engagements, or interactions with the content by liking it, sharing it, or commenting on it [13, 28–35]. For example, viewing influencer content increased knowledge, more favorable attitudes, and greater intentions to vaccinate for HPV [35]. Influencer campaigns appear able to impact health attitudes and behavior [17, 32, 34]. Self-reported exposure to flu vaccination influencer posts was associated with greater likelihood of having been vaccinated [34]. Influencer posts can drive traffic to websites [31, 34], which can help direct people to evidence-based, high-quality health information. Finally, influencer campaigns may be advantageous for reaching populations that have been harder to reach with traditional media [36].

### The present study

The goal of this pragmatic proof of concept study was to test the feasibility of collaborating with nano- (≈1000–10 000 followers) and micro-influencers (≈10 000–100 000 followers) to create and deliver CRC screening messages. Due to resource constraints, we were limited to recruiting a relatively small number of influencers (16) who would agree to take part for ≤$500 each. Working with a constrained budget aligned with our overall goal of testing a process of identifying and collaborating with influencers that could be implemented by organizations that are inexperienced with using influencer marketing and that might have few resources for communication, such as departments of health or modestly-sized health care organizations. The study focuses on addressing the challenge of reaching audiences with health messages, as awareness is a necessary precursor to behavior change. Our research questions were: (i) Is it possible to engage a large audience with CRC screening messages by collaborating with influencers to create and post brief videos? (ii) Is the cost of an influencer-based campaign comparable or lower than using other channels? (iii) Are responses to influencer-created CRC screening promotion messages positive or do they elicit resistance? (iv) As part of our proof-of-concept strategy, we also explored whether written instructions could guide influencers to produce videos consistent with theory-based message strategies, in this instance consistent with the Extended Parallel Process Model (EPPM) [37]. EPPM predicts that if both perceived threat (high perceived susceptibility and severity) and efficacy (believing one is capable of engaging in behaviors that will effectively reduce a threat) are high, people engage in danger control, that is taking protective action, but when threat is high and efficacy is low, they shift into fear control, responding with defensive reactions such as denial or message dismissal rather than behavior change. The selection of theoretical frame was somewhat arbitrary, as our goal was testing whether it was possible to shape influencer content with guidelines based on *any* theory. We chose EPPM because it lends itself to distinctly different instruction conditions (i.e. fear appeal, efficacy enhancing or both). In addition, we did not want to reduce screening motivation with any of the conditions. Despite the field’s debate over whether fear appeals can backfire, evidence indicates that they are often effective [38]. For example, in a comparison of five videos operationalizing different communication strategies, fear and self-efficacy videos resulted in the strongest screening intentions compared to an attentional control video [39]. According to EPPM, the combination of the two messages should be the most motivating. As opposed to when threat is high and efficacy is low and people are expected to engage in defensive fear control, when both threat and efficacy are high, people should engage in danger control and taking protective action [37]. Findings support the effectiveness of combining fear and efficacy messages [40], but they can also increase health behavior motivation on their own [38, 41].

## Methods

### Influencer recruitment and selection

To test strategies for recruiting and collaborating with influencers that are relatively low-resource-intensive, we used a subscription service to identify influencers rather than search for them ourselves, sought out influencers willing to produce videos for <$500 each, and provided written guidance. We used the social media influencer marketing campaign platform, Influencer Hero [42], to identify 16 influencers to take part in the campaign. Influencer Hero and similar platforms identify influencers to populate their website by scraping publicly available data. Inclusion criteria were: influencer was located in the US and spoke English, 1000–50 000 Instagram followers, ≥40% of followers were aged 45–65+, and most recent post was within 1 month. Influencers were not selected based on the topics of their posts; they focused on a range of topics including fashion, cooking, music, cute pets, and wellness. Of 1907 outreach emails sent to prospective influencers, 72 expressed interest, 52 entered negotiations, 22 signed agreements, and 16 ultimately posted videos. Emails that disclosed compensation and project details at the outset proved most effective, yielding completed posts from 2.0% of recipients, versus 0.7% for emails omitting compensation information. Influencer fees ranged from $200 to $500 (M = $389). We targeted nano- and micro-influencers due to resource constraints. However, they can be as, or more influential than macro- and mega-influencers, because they are perceived as more authentic [43]. Micro-influencers also foster more engagement than macro-influencers [44], perhaps indicating a deeper social connection with the influencer that could contribute to message persuasion. Additional description of the recruitment and collaboration process is provided in a separate manuscript (https://preprints.jmir.org/preprint/95219). The final 16 influencers had 5700 to 50 600 followers (one influencer grew their followers by 600 between eligibility screening and posting). On average, 52% (SD = 7%) of their followers were 45 years old or greater and 84% (SD = 15%) were female. Identifying information about the influencers and links to their video posts are available upon request.

### Collaborative content creation

We emailed the influencers written guidance regarding required and recommended message content (see supplemental materials). We chose this low-effort approach to maximize real-world generalizability, reasoning that most public health organizations would not be able to afford alternatives such as hiring influencer marketing strategists, hosting extensive training, or taking time to build relationships with influencers. To explore whether the written instructions could guide influencers to produce videos consistent with theory-based message strategies, they were randomly assigned to receive one of four versions of our written instructions, which included information about CRC and CRC screening and suggestions for operationalizing their assigned psychological strategy (Supplemental Materials). For the threat condition, influencers were given suggested content to increase perceived susceptibility (e.g. CRC is common statistic) and perceived severity (e.g. mortality is high statistic). In the efficacy condition, they were given content to increase perceived self-efficacy (e.g. modeling, reframing anxiety) and response efficacy (e.g. screening effectiveness statistic). The combined condition included both, and the information-only condition included neither, and influencers were instructed to deliver the required CRC information, but to avoid the use of emotion-evoking content and the use of strategies such as humor, encouraging language, or threat-arousal. Beyond incorporating the psychological and CRC elements, influencers were encouraged to create a video consistent with their brand and tailored to their followers.

The study was conducted using Instagram. Influencers were required to post a brief video to their account (i.e. posted as an Instagram Reel) where it remains for the life of the account unless removed. They were also required to share this video post to their Story, which appears at the top of followers’ feeds but disappears after 24 hours. We selected Instagram because even though it is the second most commonly used social media site among adults aged 45–75 next to Facebook, for brief videos (Reels) it offers better video engagement and return on investment [45]. As required by Instagram guidelines, they were reminded to disclose that their post was a paid partnership. Before publication, we reviewed influencers’ videos and associated post text to verify accuracy of the CRC information shared. In two instances, we requested edits to the videos. One influencer used the phrase, “early colorectal cancer screening and early colonoscopy” and was asked to remove ``early'', and the second used a backward instead of forward slash in their Bitly website address, their short-form, personalized link to the study website. We did not review for and request edits to framing because the study was designed to test whether influencers would create theory-consistent posts based on written instructions (i.e. not written instructions + coaching).

### Campaign website

While the primary goal of this campaign was to encourage the audience to be screened for CRC, we also included a link to a campaign website with additional CRC information for ethical reasons. Influencers were asked to share a website containing CRC screening information using a unique clickable Bitly link in their Instagram Story as a clickable sticker and verbally in their video. The researcher-created website consisted of 4 pages that provided an overview of CRC screening (screening recommendations, facts about screening, health care coverage and low-cost options, targeted information for people younger than 45) [46]. Given that we would be reaching people under 45 years and that rates of CRC have been increasing among people under 50 years, we included information for people younger about risk factors for early-onset CRC, CRC symptoms, and a link to a video about CRC in young people. Campaigns advertising a product are often measured by their click-through rate to the product website. However, for this study, click-throughs to the website were not a success metric because the campaign’s main call to action was to promote screening, and the website was informational only and did not include navigation to screening services. In addition, Instagram Reel video posts do not allow clickable links within the video itself, meaning that some video viewers may not have seen the Story link sticker.

### Measures

#### Influencer post engagement metrics

One month after each influencer posted their video, we recorded publicly viewable metrics for Reel views, likes, and shares, and we compiled comments, excluding those made by the influencers. Instagram measures video views as the number of times the video starts to play or replay. Video engagement rate (total likes + comments + shares)/total views) was calculated at 1 month using publicly available metrics. To provide a conservative estimate of campaign-related engagement, we excluded comments generated by one influencer’s explicit verbal prompt to comment with a song they never get tired of listening (a typically used trope of theirs, but unrelated to CRC), but we did not exclude occasional non-CRC comments on other posts because they were infrequent and not prompted by the influencer.

#### Campaign website engagement

Each influencer used a unique Bitly link, an abbreviated, customizable link with which we were able to track total clicks to the website for each influencer.

### Data analyses

#### Comment content analysis

To gauge resistance to the messages, we performed a content analysis of viewer comments. Of the 360 comments received, we analyzed a final set of 326 (34 were not publicly viewable). We classified comment sentiment as positive, negative, or neutral and used a bottom-up approach to identify a set of comment content codes. Comments were coded by two coders, and discrepancies were resolved through discussion between the two coders and the first author.

We examined whether influencers applied their assigned template (e.g. threat, efficacy, threat and efficacy, information only) when they created their video. Two raters, blind to assigned condition, classified the videos into the four message strategy categories. Disagreements were resolved through discussion.

### Quantitative analyses

We calculated frequencies for outcome metrics. We compared post engagement and website engagement in the four framing groups with descriptive statistics.

## Results

### RQ1: Is it possible to engage a large audience with CRC screening messages?

Engagement after 1 month is presented in Table 1. The 16^1^ videos garnered 89 764 video views (M = 5610, SD = 5545). In response, viewers made 360 comments, excluding 168 answering the question about a favorite song. We also excluded 219 comments made by the influencers themselves. The videos were liked 2 973 times (M = 186, SD = 145) and shared 141 times (M = 9, SD = 15). Mean video engagement rate was 7.9% (SD = 8.4%).

**Table 1:** Engagement and clicks on video link 1 month post publication.

Influencer	Assigned condition	Followers when recruited	Views	Likes	Shares	Comments	Total public engagements	Public video engagement rate	Website clicks
1	Threat	24 700	17 000	412	1	37	450	2.6%	19
2	Threat	40 000	8854	59	3	1	63	0.7%	23
3	Threat	18 000	3171	137	23	17	177	5.6%	22
4	Threat	35 000	1711	50	0	31	81	4.7%	5
Threat total/mean		117 700	30 736	658	27	86	771	3.4%	69
5	Efficacy	50 600	2361	38	58	8	104	4.4%	0
6	Efficacy	8300	1241	59	1	5	65	5.2%	5
7	Efficacy	27 100	4303	336	16	25	377	8.8%	16
8	Efficacy	21 000	703	20	1	1	22	3.1%	40
Efficacy total/mean		107 000	8608	453	76	39	568	5.4%	61
9	Threat & efficacy	9800	15 700	341	18	25	384	2.4%	1
10	Threat & efficacy	42 000	9544	380	0	12	392	4.1%	15
11	Threat & efficacy	41 000	12 100	262	10	8	280	2.3%	4
12	Threat & efficacy	6000	2146	122	2	10	134	6.2%	0
Threat & efficacy total/mean		98 800	39 490	1 105	30	55	1 190	4.2%	20
13	Information	38 000	1968	197	1	118	316	16.1%	33
14	Information	17 000	8087	395	0	10	405	5.0%	29
15	Information	5700	505	50	4	30	84	16.6%	1
16	Information	35 000	370^a^	115	3	22	140	37.8%	19
Information total/mean		95 700	10 930	757	8	180	945	18.9%	82
Grand total		419 200	89 764	2 973	141	360	3 474	7.9%	232

Note. All influencers provided a link to the website in their 24-hour story share; however among those who had 0 or 1 website clicks, two did not provide the link to the website either verbally or in associated post text and one provided an incorrect link in the associated post text. ^a^View count for this influencer was calculated at 4 months after posting due to technical difficulties accessing the count; however, most views are typically within a week of posting.

The posts elicited 232 visits to the website. Four influencer posts yielded 0 or 1 website visit, with a single visit likely a click by the influencer themselves. Among the creators of these posts, two did not provide the link to website either verbally or in the associated post text, and one provided an incorrect link in the associated post text (despite being asked to correct it).

### RQ2: What is the cost of an influencer-based campaign?

The total cost for the campaign, including payments to Influencer Hero, the influencer campaign platform ($1647), influencers, including payment platform fees ($6442), project manager who ran the campaign ($3608 for 111 hours of effort), and a URL tracking tool ($120), was $11 817. Video views therefore cost $0.13 each at the 1-month mark. We excluded research costs (e.g. analyzing outcome data, second reviewer of draft videos) as these would not be incurred by an organization considering running an influencer campaign.

### RQ3: Are responses to influencer-created CRC screening promotion messages positive or do they elicit resistance?

The 326 coded comments were overwhelmingly positive (96%), and the remaining were neutral (4%). There were no negative comments (Cohen’s kappa for sentiment = 0.95). Some comments consisted only of emojis (10%), others were related to the influencers themselves (6%) or were unclear (1%), but most related to CRC (83%) (Cohen’s kappa for comment topic = 0.88). Among those related to CRC, 79% reiterated the importance or value of the CRC message or expressed appreciation for the message (e.g. “Such an important message. This needs to be shared loud and wide. Thank you!”). In 20% of the comments, viewers mentioned their own screening status or experience (e.g. “I get screened every 5 years.”). A few viewers mentioned other people’s experiences with screening or CRC (6%), perceptions of their risk for CRC (3%), that they learned something new from the video (4%), or remarked on young people getting CRC (4%). A small number of people (4%) made informational statements (e.g. “The survival rate is good (if) caught early”). All informational statements were accurate. The comment content codes were not mutually exclusive, so we calculated Cohen’s kappa for the eight codes that were used >10 times. Values ranged from 0.63 to 0.92 (M = 0.81).

### RQ4: Can written instructions guide influencers to produce videos consistent with theory?

We piloted whether text-only instructions could guide influencers to produce theory-based message content. Actual and coded categories were consistent in 13 of 16 videos (81%). There was one error of omission, where the influencer was assigned to threat and efficacy but did not include efficacy elements. The other two mismatches were influencers who added elements not assigned to them. One added efficacy-promoting elements to what should have been a threat-only message, and the other added threat and efficacy elements to what should have been an information-only message.

## Discussion

A collaborative campaign with 16 nano- and micro-social media influencers resulted in nearly 90 000 video views. There was no evidence of resistance to the messages; engagement was high, and reactions were positive. Influencers were able to include efficacy or threat-promoting elements when asked to do so in written instructions.

This study builds on a number of prior studies that have tested the feasibility and efficacy of collaborating with social media influencers to disseminate health messages [13, 28–33, 35]. This is the first study, to the authors’ knowledge, to test a campaign to promote cancer screening; prior campaigns have promoted vaccination, pediatric health, awareness of and support for harm reduction, or mental health support. The campaign topic appears to have been well-received by influencers’ audiences, with positive comment sentiment and an above-average video engagement rate. The engagement rate of nearly 8% is a conservative estimate as it excludes comments made by the influencers and includes only publicly viewable engagements (likes, comments, shares). Although no standardized benchmark for Instagram Reel engagement rates exists, most industry blogs suggest averages around 2%–3% [47], with some estimating rates closer to 6%–7% [48]. None of the comments were negative, which was also noted in another health campaign [28]. None of the comments spread misinformation. In the present study, views cost $0.13. One would want to be cautious about comparing campaign costs in a rapidly changing media environment. In a large social media ad campaign conducted in 2025 in Singapore, views cost about $0.11 each [49]. In contrast, the cost of television ads, e.g. could be millions of dollars [50]. A television and radio campaign to prevent youth smoking initiation cost $41 (1996 USD) per child viewer [51].

We used an online influencer marketing campaign platform to identify influencers and purposefully recruited influencers who create content on a range of topics, including non-health topics, to increase the likelihood of reaching individuals who may not be highly health-conscious and already CRC screening adherent. Especially if one is trying to target a narrow audience and is trying to recruit niche influencers, alternatives such as contracting with a marketing firm to search for influencers [35] or a manual search [13] might be necessary, but these approaches could be challenging for resource-limited organizations. We attempted to guide influencers to follow theory-based (EPPM) guidelines. This was largely successful; influencers were able to create both threat and efficacy promoting content and the majority followed the guidelines for their assigned strategy. Two of three mismatches were influencers who added threat or efficacy elements, which are likely intuitive strategies, especially given that many people have now grown up being exposed to health campaigns that deploy these strategies. Brief written instructions may be adequate for guiding influencers. One study found no benefits of more intensive influencer training; participation in a day-long training did not increase the percentage of influencers’ posts containing evidence-based mental health information. In comparison, there was a small increase (31% versus 28%) for influencers receiving materials to review on their own [13]. Perhaps even more important than determining whether influencers follow theory-based guidance is whether providing this guidance improves engagement or message effectiveness and reduces misinformation. On one hand, too many or too narrow requirements may stifle influencer creativity. On the other, without some broad guidance, influencers may default to the kinds of messages that seem most intuitive, but could be counterproductive (e.g. threat-only messages). Attempting to review and correct videos after they are completed is not necessarily practical, as, in our experience, influencers are reluctant to make edits to their posts given the effort involved.

### Public health implications

Public health organizations could benefit from partnering with influencers to co-create and disseminate messaging campaigns. One of the most attractive features of working with influencers is its relatively low cost compared to traditional media such as television [50]. Social media ads are not as expensive, but they can have significant up-front content development costs and typically require working with a marketing agency to develop the campaign. A second benefit of influencer campaigns is that they reduce risk through diversification. When an organization works with a single marketing agency to develop a campaign, there is only one opportunity to create a well-received and effective campaign. Working with a large number of influencers means the campaign is more heterogeneous, creating multiple opportunities to develop engaging posts. Public health organizations may have a few concerns about partnering with influencers. They may be concerned that influencer campaigns are only suitable for national audiences. Although our goal was not to develop a regional campaign, there are influencers with strong regional followings who could contribute to campaigns targeting a defined geographic area such as states [32], or counties [29]. Organizations may be concerned that it would be challenging to create an evolving, longer-term campaign. While we tested one-time posts, others have shown it is also possible to contract with influencers to post multiple times [28]. Finally, organizations might be concerned that in working with influencers, it will not be possible to use evidence-based communication strategies. In the present study, it was possible to guide influencers to include EPPM-based message components.

### Strengths and limitations

A major problem facing health communicators today is a crisis of access to audiences. Media exposure has shifted from traditional to social media. The main focus of this study was therefore addressing this challenge, and results indicate that it is possible to gain exposure to large audiences by collaborating with social media influencers. We did not assess viewers’ CRC screening behavior, but penetrating the information environment is a necessary precondition for later cognitive, attitudinal, or behavioral effects. If the goal were to fine-tune how to collaborate with influencers to optimize message persuasion and health behavior change, this might be best addressed with a less pragmatic paradigm such as controlled laboratory experiments. The aspect of the study where we randomized influencers to receive guidance on using a theory-based strategy is the first step toward testing the use of health behavior theory in social media collaboration. Subsequent steps might include testing whether influencer posts are more engaging and effective if they use theory-based strategies and comparing alternative theory-based strategies.

We were unable to assess reach, the number of unique viewers who saw the post, or whether viewers watched the entire video, as these data are not publicly available and we could not reliably obtain this information from the influencers themselves. The scope of the study was limited; a larger study could reveal additional challenges in recruiting and communicating with influencers and would allow us to test whether some message strategies elicit more engagement than others. An exploratory goal of the study was to pilot if providing guidelines that were low burden on influencers and the client could encourage theory-based strategies. A larger sample of influencers would be needed to rigorously test this research question. Finally, social media influencer campaign studies that assess behavioral outcomes are a needed next step.

## Conclusion

Collaborating with social media influencers is a feasible and relatively low-cost strategy for disseminating CRC screening messages to the public. Even influencers with fewer than 50 000 followers generated substantial numbers of views, suggesting that such campaigns can prompt information seeking and potentially influence preventive health behaviors. Viewers held positive views of the video content, and comments did not promote false information. In summary, partnering with social media influencers is a promising way to expand the reach of health messages. Although the partnership process requires staff time, influencer campaigns offer the possibility of creating innovative and impactful messages.

## Supplementary Material

Supplemental_materials_Influencer_Guide__cyag024

## Footnotes

1 One influencer’s view count was calculated at 4 months following posting due to technical difficulties accessing the count; however, most views are typically within a week of posting.
