# Supplementary material for: Feasibility and cost of partnering with social media influencers to promote colorectal cancer screening
Source: Health Educ Res. 2026 Aug 21;41(5):cyag024. doi: 10.1093/her/cyag024 (PMC13494905; doi:10.1093/her/cyag024)
Supplement: Supplementary file 1 [file supplemental_materials_influencer_guide__cyag024.pdf]

UNIVERSITY AT BUFFALO COLORECTAL CANCER SCREENING PROJECT

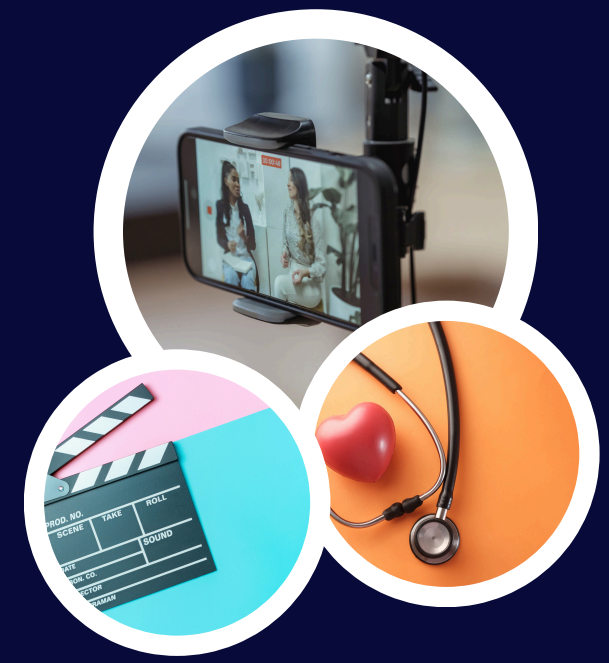

CONTENT GUIDE

FOR SOCIAL MEDIA CREATORS

WHO ARE WE?

Hi, we're Heather and Natasha and we are researchers at the University at Buffalo. We're working on a project trying to encourage people to get screened for colorectal cancer because early detection can save lives. We're excited to work with you to get the word out about this important topic. If you'd like to learn more about our backgrounds and published research, please email ncallard@buffalo.edu

WHAT ARE WE ASKING YOU TO DO?

Please create 1 video promoting colorectal cancer screening (using the provided facts and strategies) that is likely to engage your audience.You will send it to us for review before posting (along with the copy you will use). There are no length requirements but it should be consistent with your usual content. Please post the video as a Reel (1x) and share it to your Story (1x).

We want to work with you because we like your style and creativity and a large proportion of your followers are 45-75 years old. Keeping this audience in mind when creating your video is great.

WHAT INFO SHOULD BE IN THE VIDEO?

Please make the video as authentic and engaging as possible! We leave the creativity up to you, but there are three things that must be incorporated:

1. Be factual (check with us if you use facts other than those we provide)
2. Must include a FEAR AND SELF-EFFICACY appeal (learn more about what we mean on the next page)
3. Do not include humor. We are using this strategy in other videos.

UNIVERSITY AT BUFFALO COLORECTAL CANCER SCREENING PROJECT

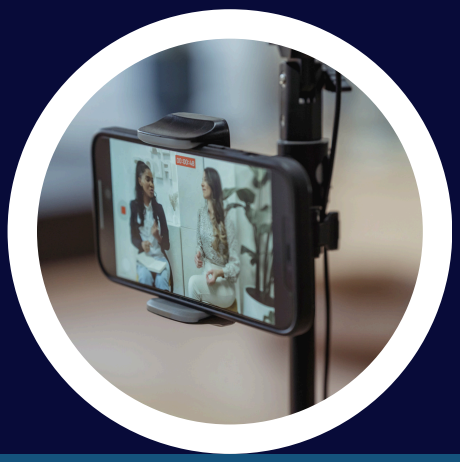

THE FACTS TO DISCUSS

Below are some facts about colorectal cancer screening. We ask that you work in several of these facts, but you do not have to include them all. It is more important that the video is sincere and consistent with your style than that it include all the information below. If you want to add other information, please check with us first.

Key points:
·Screening is recommended for people 45-75
·colorectal cancer affects women and men
·screening can prevent cancer
·screening can be affordable
·screening does not have to be invasive
·Most colorectal cancers are not due to inherited risk.

What is colorectal cancer screening?
The goals of colorectal cancer screening is to detect pre-cancerous growths before they become cancer or detect cancer early when it is easiest to treat.

Why should people get screened?
Screening can find and remove small growths called polyps in the colon or rectum before they turn into cancer. Early detection can mean more treatment options and better outcomes.
Colorectal cancer is often symptomless. Most people who have pre-cancerous polyps or early stage colorectal cancer do not have symptoms. Therefore, screening is important for catching cancer before is starts, or when it is most treatable.

There are two main kinds of colorectal cancer tests:
Home tests check the stool for signs of cancer. These tests are less invasive and easier to have done, but they need to be done annually or every three years depending on the type of test. If your home test is positive you will need to have a colonoscopy.
Colonoscopies look at the structure of the colorectal and rectum for any abnormal areas. They are often done every 10 years.

CALL TO ACTION (verbally say out loud at least once and include in copy):
VISIT bit.ly/EGCRC2025 to learn more.

BE SURE TO INCLUDE A FEAR & A SELF-EFFICACY APPEAL

For this video, we are specifically testing if a “fear appeal”, or making people worried and anxious, followed by a “self-efficacy appeal”, or making people feel confident that the screening tests are effective and easy to complete, can result in them wanting to be screened. Present fear/anxiety evoking information first.

Here are some options of what you could say:

FEAR

- Colorectal cancer is the second most common cause of cancer deaths in the United States.
- About 1 in 24 men and 1 in 26 women will develop colorectal cancer in their lifetimes.
- Nearly 50,000 people die of colorectal cancer every year in the US.
- Deaths have been increasing for people under 55.

SELF-EFFICACY

- Colonoscopy screening could prevent up to 88% of deaths from colorectal cancer.
- Talk about a friend or family member who got tested and felt relieved and proud of themselves after doing it.
- Talk about a friend or family member who got tested using a home test and told you it was simple and quick.
- It is normal to feel anxious about screening for cancer but that’s our way of our body tell us that it is an important step to protect ourselves and even our families.

UNIVERSITY AT BUFFALO COLORECTAL CANCER SCREENING PROJECT

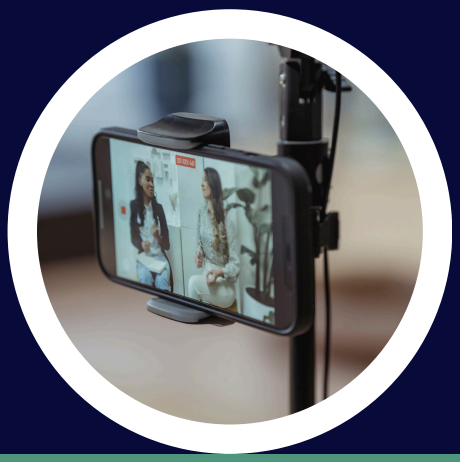

Recap:

1. **Please create a video consistent with your usual style and length**
2. **Work in some colorectal cancer screening facts we provided**
3. **Include the specific type of appeal we described**

Next steps:

1. **Send us the video for approval**
2. **Upon approval, post once as a Reel and share once as a story (please do not share more than this until after 1 month to help us evaluate a standardized approach)**
3. **Send us all engagement statistics for both the Reel and story at: 1 day (24 hours), 1 week (7 days), and 1 month (30 days)**
4. **We will complete payment through the agreed upon method**

Thank you so much for collaborating with us on this important health project! So we can evaluate what messaging approaches are most persuasive, we ask you to please follow the guidelines we presented closely. We provided a lot of information, so please don’t hesitate to reach out with any questions. We would be happy to schedule a quick phone call or Zoom meeting to talk through any ideas as well. Overall, be yourself and engage your audience with these important messages! Thank you!

Contact us:
Natasha Allard
ncallard@buffalo.edu
